# PD-L1 expression is a predictive biomarker for CIK cell-based immunotherapy in postoperative patients with breast cancer

**DOI:** 10.1186/s40425-019-0696-8

**Published:** 2019-08-27

**Authors:** Zi-Qi Zhou, Jing-Jing Zhao, Qiu-Zhong Pan, Chang-Long Chen, Yuan Liu, Yan Tang, Qian Zhu, De-Sheng Weng, Jian-Chuan Xia

**Affiliations:** 1 Collaborative Innovation Center for Cancer Medicine, State Key Laboratory of Oncology in South China, Sun Yat-sen University Cancer Center, Guangzhou, 510060 People’s Republic of China; 2 Department of Biotherapy, Sun Yat-sen University Cancer Center, Guangzhou, China

**Keywords:** Adjuvant CIK immunotherapy, Breast cancer, Immunohistochemistry, PD-L1, Prognosis

## Abstract

**Background:**

A sequential combination of radiochemotherapy/endocrinotherapy and cytokine-induced killer cell (CIK) infusion has been shown to be an effective therapy for post-mastectomy breast cancer based on statistical analysis of the patient population. However, whether an individual could obtain an improved prognosis from CIK cell-based treatment remains unknown. In the present study, we focused on immune microenvironment regulation and specifically investigated the relationship between PD-L1 expression and survival benefit from CIK immunotherapy in breast cancer.

**Methods:**

A total of 310 postoperative breast cancer patients who received comprehensive treatment were enrolled in this retrospective study, including 160 patients in the control group (received chemotherapy/radiotherapy/endocrinotherapy) and 150 patients in the CIK cell treatment group (received chemotherapy/radiotherapy/ endocrinotherapy and subsequent CIK infusion).

**Results:**

We found that overall survival (OS) and recurrence-free survival (RFS) were significantly better in the CIK group than that in the control group. PD-L1 expression in tumor tissue sections was showed to be an independent prognostic factor for patients in the CIK treatment group using multivariate survival analysis. Further survival analysis in the CIK group showed that patients with PD-L1 tumor expression exhibited longer OS and RFS. In addition, among all patients who were enrolled in this study, only the patients with PD-L1 expression experienced survival benefits from CIK treatment.

**Conclusions:**

Our study showed the relationship between PD-L1 expression and CIK therapy and revealed that PD-L1 expression in the tumor is as an indicator of adjuvant CIK therapy for postoperative breast cancer.

**Electronic supplementary material:**

The online version of this article (10.1186/s40425-019-0696-8) contains supplementary material, which is available to authorized users.

## Background

Breast cancer is a type of malignant neoplasm that occurs in the glandular epithelium and has the highest incidence among female tumors [1]. At least 400,000 women die from breast cancer annually all over the world, accounting for 14% of the total cancer-related deaths [2]. The incidence of breast cancer in China is relatively lower than that in countries in North America, Australia or New Zealand [3, 4]. However, the absolute number of deaths from this disease is still enormous due to the large population base [3]. Therapies for breast cancer include surgery, chemotherapy, radiotherapy, endocrine therapy and bio-targeted therapy [5–7]. Despite enormous improvements in these treatment modalities in the past 20 years, the prognosis of breast cancer is still not ideal [8]. Therefore, the exploration of more effective treatments for breast cancer is necessary and pressing.

Cytokine-induced killer (CIK) cells, a group of heterogeneous cells that are harvested from in vitro culture, are stimulated with a variety of cytokines (such as anti-CD3 monoclonal antibodies, IL-2 and IFN-γ) [9–11]. CIK cells exhibit many excellent characteristics, including rapid proliferation, enhanced anti-tumor activity and a broader spectrum of anti-tumor activity (more sensitive to multidrug-resistant tumor cells and cancer stem cells) [12, 13]. In addition, CIK cells is a cohort of autologous cells that is safe for clinical application [14]. A series of studies has shown that CIK-based treatment could significantly improve the prognosis of both hematologic malignancies and solid tumors, including breast cancer [15–21]. However, not all tumor patients who receive CIK cell infusion exhibit improved outcomes; some patients are nonresponsive. Therefore, we sought to investigate what methods can identify patients who are suitable for CIK cell treatment. As an immunotherapy, CIK-based treatment aims to activate and enhance the body’s immune system to improve its anti-tumor ability, which is intrinsically a type of immune regulation [12, 17]. In turn, the activation of infused CIK cells will also be affected by the immune microenvironment in vivo [22, 23]. Thus, we aimed to explore whether immune factors are correlated with the clinical efficacy of CIK treatment among individuals.

Programmed death-ligand 1 (PD-L1; B7-H1 or CD274) plays an important role in immunosuppression and immune escape [24]. When bound to its ligands programmed death-1 (PD-1) and B7.1 (CD80), PD-L1 could mediate T cell inactivation by preventing T cell activation, migration, proliferation and secretion [25]. Many studies have indicated that PD-L1 overexpression is a poor prognosis biomarker in many cancer types and is related to tumor metastasis and recurrence [26–29]. However, a series of recent studies have confirmed that higher PD-L1 expression in tumor tissue intrinsically reflects a stronger ongoing anti-tumor immune response in the body [30, 31]. In addition, tumor patients with tumors overexpressing PD-L1 have been confirmed to benefit most from cancer immunotherapy [32, 33]. Our previous study had also revealed that positive PD-L1 expression could predict the efficacy of CIK cell treatment for patients with hepatocellular carcinoma (HCC) [34]. However, whether this relationship between PD-L1 expression and survival benefit from CIK infusion is present among patients with breast cancer remains unclear.

In this study, we performed a retrospective analysis to clarify the efficacy of CIK cell immunotherapy after comprehensive treatment in postoperative breast cancer patients. Importantly, we aimed to explore whether PD-L1 expression could function as a biomarker for adjuvant CIK treatment among breast cancer patients.

## Methods

### Patient population

Between December 1, 2009, and December 31, 2013, the medical records of patients with breast cancer from a computerized database in the Sun Yat-Sen University Cancer Center (Guangzhou, China) were reviewed. This database recorded clinicopathological information of the patients at recruitment, including details about age, menopausal status, tumor characteristics, TNM (tumor–node–metastasis) staging, treatment and outcome. All the female patients underwent surgery, including quadrantectomy or mastectomy and axillary lymph node dissection. Subsequently, most patients received chemotherapy, radiotherapy or endocrinotherapy depending on their clinical stage. Following termination of the normal comprehensive treatment, a subpopulation of the patients of informed consent received at least four cycles of CIK immunotherapy if they had no post-operative dysfunction in any organ, no systemic immunosuppressive therapy, no active autoimmune disease, and no occurrence of serious adverse events during CIK cell immunotherapy. For further selection, random number table method was then employed to select patients for satisfying the sample size requirements of the control group and the CIK treatment group. Patients were excluded from the study based on the following criteria: the presence of a distant metastasis at diagnosis, a history of other malignancies, treatment with neoadjuvant chemotherapy/radiotherapy, patients who did not receive any chemotherapy/radiotherapy/endocrinotherapy after mastectomy and patients who received CIK treatment after recurrence. After review, 310 patients met the study criteria and were included for further analysis. Among them,150 patients had received CIK treatment (CIK group), whereas the other 160 patients did not receive CIK treatment and were thus enrolled into the control group for comparison.

### Follow-up

After surgery, all patients underwent regular follow-up at our outpatient department. General follow-up was required every 3 months in the first 2 years, every 6 months in the following 3 years and then annually thereafter. The follow-up in the outpatient department included a comprehensive evaluation of clinical and laboratory parameters. Chest CT/MRI was performed when recurrence or metastasis was suspected. Recurrence-free survival (RFS) was defined as the time from the definitive surgery to the time of the first recurrence (local or distant) or the last follow-up. Overall survival (OS) was defined as the time from surgery to the time of death from any cause or the date of the last follow-up.

### Generation of CIK cells and treatment

The generation and application of autologous CIK cells for treatment were performed according to the established procedures [35]. Briefly, 2 weeks after the patients had completed comprehensive treatment and when routine blood examination had returned to normal, a sample of heparinized peripheral blood (50–60 mL) was collected. Peripheral blood mononuclear cells (PBMC) were sorted with Ficoll gradient centrifugation followed by suspension in X-VIVO 15 serum-free medium (Longza, Shanghai, China). In culture, Recombinant Human Interferon-γ (rhIFN-γ; 1000 U/mL; Clone-gamma, Shanghai, China) was added for the first 24 h followed by the addition of anti-human CD3 monoclonal antibody (100 ng/mL; R&D Systems, Minneapolis, USA), Recombinant Human Interleukin 2 (rhIL-2; 1000 U/mL; Beijing Sihuan, China) and Recombinant Human Interleukin-1α (IL-1α; 100 U/mL; Life Technologies, Waltham, USA) for the induction of CIK cells. During culture, fresh medium with rhIL-2 (1000 U/mL) was typically added, and the cell density was maintained at 2 × 10^6^ cells/mL. The CIK cells were harvested on the 14th day. Before infusion, a series of necessary quality examinations were conducted, including cell count, viability and phenotypic analysis and the detection of possible contamination. Approximately 50 to 60 mL of peripheral blood was obtained from the patient prior to infusion for the preparation of CIK cells to be used in the next treatment. Then, the harvested autologous CIK cells that are free of microbial contamination were transferred to the patients by intravenous infusion within a 30-min period. Patients generally received CIK cell infusions for at least 4 cycles, with a 2-week interval between every 2 cycles. After that, if the patient was in a stable condition and requested additional therapy, additional cycles of CIK maintenance treatment were administered. However, if the disease progressed or the patients did not want to continue, the CIK cell infusion therapy would be stopped (Additional file 1: Figure S1). This retrospective study was performed in accordance with the Declaration of Helsinki and according to national and international guidelines, and was also approved by the Ethics Committee of Sun Yat-Sen University Cancer Center; the written informed consent was obtained from each patient.

### The phenotype analysis of CIK cells using flow cytometry

CIK cells were resuspended at 2 × 10^5^ cells per 100 μL of phosphate-buffered saline (PBS) and incubated for 30 min at 4 °C with the following anti-human antibodies: anti-CD3-PE-Cy5, anti-CD4-FITC, anti-CD8-PE-CF594, anti-CD25-APC, anti-CD56-PE-Cy7, anti-CD45RO-APC, and anti-CD62L-FITC (all from BD Bioscicence). The cells were analyzed using a CytomicsTM FC500 Flow Cytometer (Beckman Coulter, USA). Data analysis was performed with CXP analysis software (Beckman Coulter, USA).

### Intracellular cytokine production analysis of CIK cells using flow cytometry

CIK cells were collected and incubated at 37 °C for 6 h in X-VIVO 15 serum-free medium containing 50 ng/mL phorbol 12-myristate 13-acetate (PMA) (Sigma, USA) and 500 ng/mL ionomycin (Sigma, USA). Brefeldin A (Sigma, USA),10 ng/mL, was added for the final 5 h of incubation to block cytokine secretion. The cells were harvested, fixed with 4% paraformaldehyde for 15 min at room temperature, and permeabilized with 0.1% saponin (Sigma, USA). Finally, the cells were labeled with anti-CD8-PE-CF594, anti-IFN-γ-APC, anti-TNF-α-FITC, anti-Granzyme B-APC, and anti-Perforin -FITC and analyzed by flow cytometry.

### Proliferation analysis of CIK cells

The CellTrace CFSE Cell Proliferation Kit (Molecular Probes, Shanghai, China) was used to determin the number of active T cells according to the manufacturer’s protocol.

### Cytotoxicity analysis of CIK cells and tumor cell lines culture

The cytotoxic specificity of the CIK cells obtained from the breast cancer patients received CIK treatment was analyzed using a Cyto Tox 96 Lactate Dehydrogenase Assay Kit (Promega,USA) according to the manufacturer’s protocol. The effector cells in these tests were CIK cells and the target cells were breast cancer cell lines MCF7 which were obtained from the Committee of the Type Culture Collection of the Chinese Academy of Sciences (Shanghai, China) and cultured at 37 °C in 5% CO2 in DMEM medium (Gibco, USA) supplemented with 10% fetal bovine serum (FBS; Gibco, USA) and 1% penicillin-streptomycin. Cytotoxicity was quantified after the effector and target cells were co-incubated for 12 h at an effector cell to target cell (E: T) ratio of 3:1, 10:1, or 30:1.

### Tumor tissue samples and immunohistochemical analysis of PD-L1 expression

A total of 310 samples underwent immunohistochemical analysis of PD-L1 expression. All the tumor tissues were confirmed by pathological examination, fixed in 10% neutral buffered formalin and then embedded in paraffin. Tissue sections of 3-μm thickness were deparaffinized followed by rehydration in a graded ethanol series. For antigen retrieval, the tissues were boiled in EDTA (1 mM, pH 8.0) in a microwave oven for 15 min. Endogenous peroxidase activity was blocked by treating the tissues with 0.3% H_2_O_2_ for 10 min, and nonspecific staining was abolished by treatment with goat serum for 30 min. Slides were incubated with primary monoclonal antibodies against PD-L1 at a 1:200 dilution (Cell Signaling Technology, Danvers, USA) in a humidified chamber at 4 °C for 12 h. After washing with phosphate-buffered saline, the slides were incubated with horseradish peroxidase-conjugated secondary antibody (Gene Tech Shanghai, China) at room temperature for 30 min. Finally, diaminobenzidine tetrahydrochloride was employed to develop the positive staining, and the tissues were subsequently counterstained with hematoxylin. Then, all the slides were dehydrated.

The stained sections were evaluated by two experienced pathologists who were not informed of the patient’s clinicopathological parameters. Based on the pattern of PD-L1 expression, the percentage of tumor cells with membranous PD-L1 staining was calculated, and the specimens were divided into the ≥5% and < 5% expression cohorts. A level of ≥5% PD-L1 expression in the tumor was defined as PD-L1 positive, and a level of < 5% PD-L1 expression in the tumor was defined as PD-L1 negative. Any inconsistencies between the two researchers in the evaluation process are subject to further adjudication.

### Statistical analysis

SPSS 20.0 was used for the statistical calculations. Pearson’s chi-squared test and Fisher’s exact test were employed to compare the differences in demographic and clinical variables between the two groups of patients with breast cancer. The Mann-Whitney test was used to compare PD-L1 expression levels. The Kaplan-Meier method was employed to analyze the rates of RFS and OS among patients. The log-rank test was used to compare the differences. The Cox proportional hazards regression model was used for univariate and multivariate analyses. The results of the phenotype, intracellular cytokine production, proliferation, and cytotoxicity of CIK cells are represented as the mean ± SD and analyzed using Student’s t-test. A *p* value of less than 0.05 was defined as statistically significant.

## Results

### Patient demographics and clinical characteristics

This retrospective study enrolled a total of 310 postoperative breast cancer patients. Briefly, among all the patients, there were 165 (53.2%) with TNM stage I/II tumors and 145 (46.8%) with TNM stage III tumors. There were 109 patients (35.2%) with a <  0.21 positive lymph node ratio and 201 cases (64.8%) with a ≥ 0.21 positive lymph node ratio (Table 1). The patients were divided into two groups based on whether they received CIK cell infusion (the CIK treatment group and the control group). Specifically, in the control group, postoperative patients received conventional therapy based on their clinical conditions, including chemotherapy, radiotherapy or endocrinotherapy. In the CIK treatment group, the patients received CIK cell infusions in addition to their normal regimens. The clinicopathological parameters and comprehensive treatments between the two groups were well matched, and there were no statistically significant differences in variables such as age, positive lymph node ratio, TNM stages, pathologic grades and the expression of PD-L1 (*p* > 0.05) (Table 1).

**Table 1:** Demographics and clinical characteristics of patients in the CIK treatment and Control groups

Clinicopathologic variables	Control Group (*n* = 160)	CIK Treatment Group (*n* = 150)	*p* value
Age (y)			0.685
< 50	59	52	
≥ 50	101	98	
Tumor size (mm)			0.500
< 20	76	77	
≥ 20	84	73	
TNM stage			0.849
I	19	15	
II	66	65	
III	75	70	
Histological differentiation			0.905
I/II	96	89	
III	64	61	
Positive lymph node ratio			0.628
< 0.21	59	50	
0.21 ≤ x < 0.65	80	75	
≥0.65	21	25	
Receptor status			
ER			0.602
Positive	51	52	
Negative	109	98	
PR			0.877
Positive	75	69	
Negative	85	81	
Her2			0.604
Positive	60	52	
Negative	100	98	
PD-L1 expression			0.264
Positive	42	44	
Negative	118	106	
Chemotherapy			0.549
Yes	150	138	
No	10	12	
Radiotherapy			0.267
Yes	149	144	
No	11	6	
Endocrinetherapy			0.136
Yes	109	90	
No	51	60	
TNBC			0.952
Yes	26	24	
No	134	126	

### The phenotype of CIK cells

**Fig. 1:**
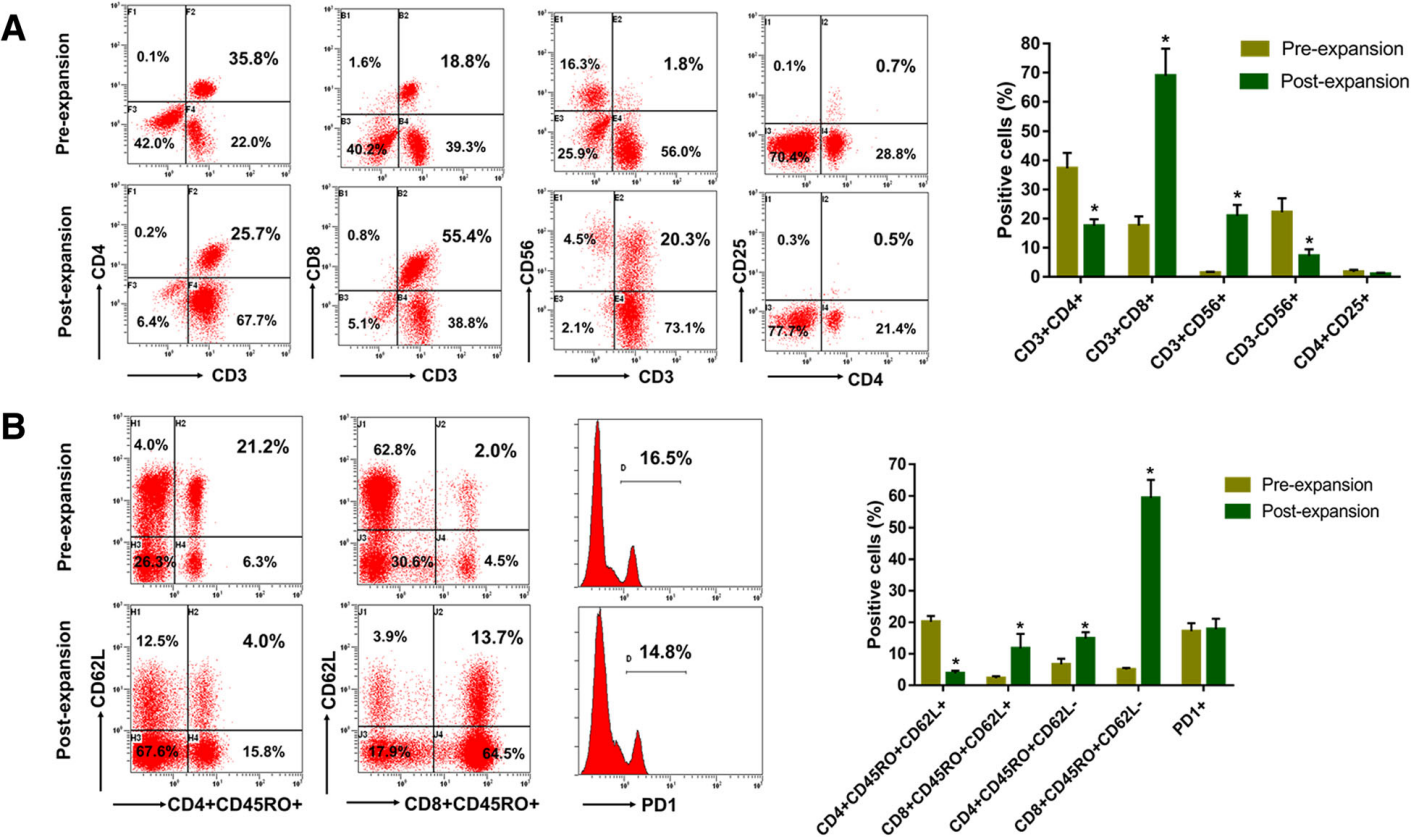
The phenotype of CIK cells in breast cancer patients before and after the expansion. **a** The percentage of CD3^+^CD4^+^ T cells, CD3^+^CD8^+^ T cells, CD3^+^CD56^+^ NKT cells, CD3^−^CD56^+^ NK cells, and CD4^+^CD25^+^ regulatory T cells of CIK cells before and after the expansion. **b** The percentage of CD4+ central memory T cells (TCM), CD4+ effector memory T cells (TEM), CD8+ TCM, and CD8+ TEM of CIK cells before and after the expansion. * *p* < 0.05

After culture and expansion, the final count of CIK cells was between 8.7 × 10^9^ and 12 × 10^9^, and the viability could be greater than 95%. The percentage of CD3^+^ T cells was ranged from 75.9 to 93.4% with a median of 87.9%, among which the percentage of CD3^+^CD4^+^ T cells was ranged from 15.3 to 21.3% with a median of 17.05%, the percentage of CD3^+^CD8^+^ T cells was ranged from 40.1 to 80.3% with a median of 67.8% and the percentage of CD3^+^CD56^+^ NKT cells was ranged from 6.1 to 57.9% with a median of 20.3%. Additionally, the percentage of CD3^−^CD56^+^ NK cells was ranged from 4.5 to 11.1% with a median of 7.0%, and the percentage of CD4^+^CD25^+^ regulatory T cells was ranged from 0.6 to 1.5% with a median of 0.95%. All the prepared cells were determined to be free from bacterial and fungal contamination. They were also negative for mycoplasma and included < 5 EU endotoxin. Then, all autologous CIK cells were infused back into the corresponding patients. Compared with the PBMC, we found that the populations of CD3^+^CD56^+^ NKT cells and CD3^+^CD8^+^ T cells of CIK cells were significantly increased after in vitro expansion (Fig. 1a). Conversely, the populations of CD3^−^CD56^+^ NK cells and CD3^+^CD4^+^ T cells of CIK cells were significantly decreased after in vitro expansion (Fig. 1a). The population of CD4^+^CD25^+^ regulatory T cells of CIK cells had no obvious change after in vitro expansion (Fig. 1a). Furthermore, we also found that populations of CD8^+^ central memory T cells (TCM, CD8^+^CD45RO^+^CD62L^+^), CD8^+^ effector memory T cells (TEM, CD8^+^CD45RO^+^CD62L^−^) and CD4^+^ TEM (CD4^+^CD45RO^+^CD62L^−^) were significantly increased after in vitro expansion, however, the populations of CD4^+^ TCM (CD4^+^CD45RO^+^CD62L^+^) was decreased after in vitro expansion (Fig. 1b). In addition, the expression of PD1 on CIK cells showed no significant change after in vitro expansion (Fig. 1b).

### The intracellular cytokine production, cell proliferation, and cytolytic activity of CIK cells

**Fig. 2:**
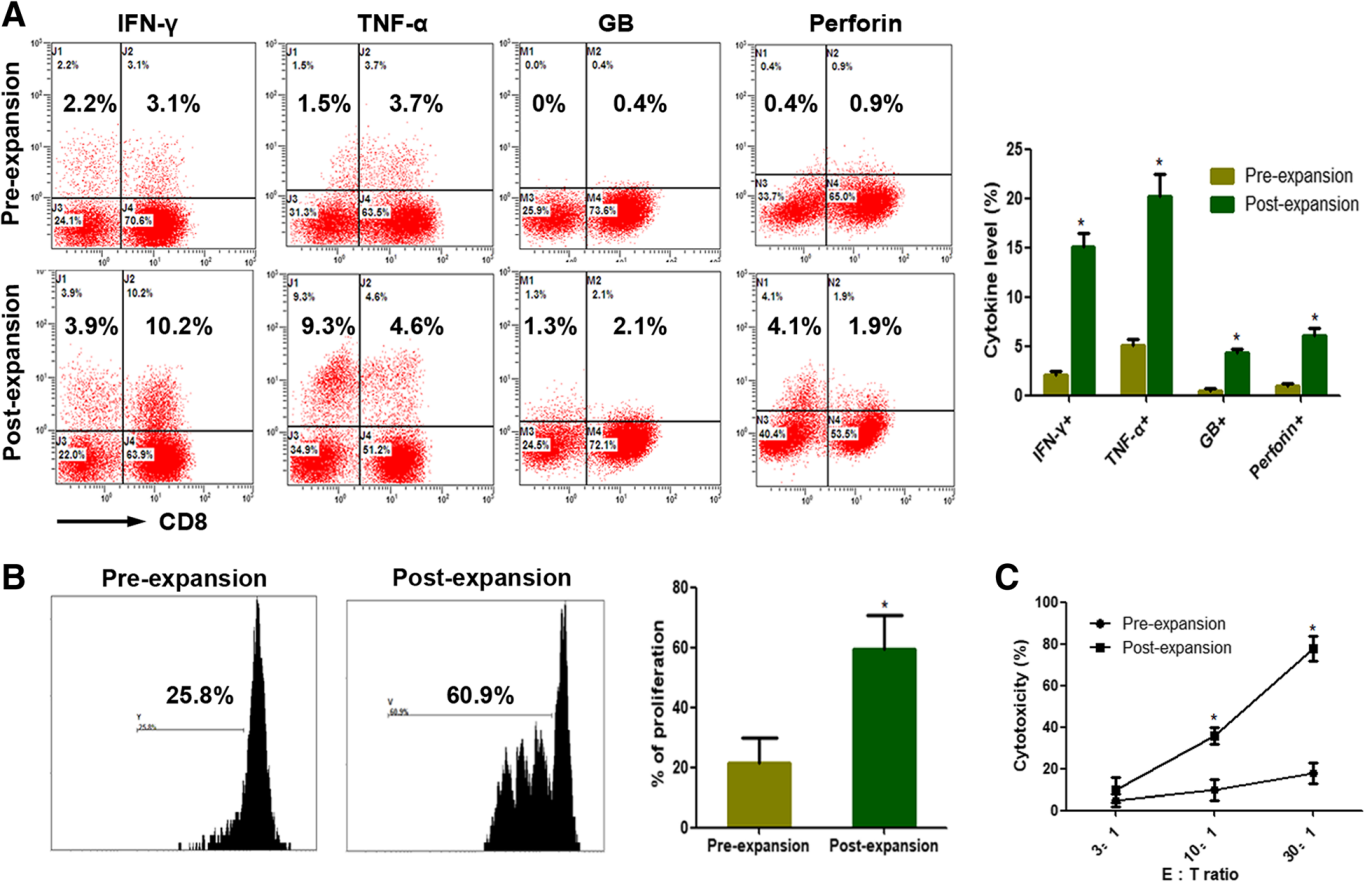
The intracellular cytokine production, cell proliferation, and cytolytic activity of CIK cells in breast cancer patients before and after the expansion. **a** IFN-γ, TNF-α, Granzyme B (GB) and Perforin production of CIK cells before and after the expansion. **b** The proliferation ability of CIK cells before and after the expansion. (**c**) The cytolytic activity of CIK cells before and after the expansion in response to MCF7 cell line, at a 3:1, 10:1, or 30:1 E: T ratio. E: T ratio, effector cell to target cell ratio. * *p* < 0.05

After culture and expansion, CIK cells secreted more amounts of cytokines, including IFN-γ, TNF-α, Granzyme B and perforin compared with the PBMC (Fig. 2a). Furthermore, the proliferation of CIK cells was significantly enhanced after in vitro expansion compared with the PBMC (Fig. 2b). As shown in Fig. 2c, for the MCF7 cell line, the cytolytic activity of CIK cells was significantly enhanced (Fig. 2c).

### Adverse events from CIK cell infusion

CIK cells therapy-related adverse events were relatively mild, mainly including fever, chill, arthralgia/myalgia, fatigue and anorexia. In our study, only 12 patients who were treated with CIK cells experienced adverse events, including 4 cases of fever (38–40 °C), 3 cases of fatigue and anorexia, 3 cases of arthralgia/myalgia, 1 case of nausea/vomiting, and 1 case of transient hypertension (Table 2). No treatment-related serious adverse events such as pneumonitis, colitis, hepatitis, and treatment-related deaths appeared in any of the patients. Median time to onset of CIK cells therapy-related adverse events was 4.5 h (range, 0.5–30.0) (Table 2). The median duration of CIK cells therapy-related adverse events was 12 h (range, 0.5–36.0) (Table 2).

**Table 2:** CIK cells therapy-related adverse events according to category and grade

Category	Patients no.(%)				Time to Onset(hours), Median (range)	Time of duration(hour), Median (range)
	Total	Grade 1–2	Grade 3–4	treatments		
Any	12(8.0)	11(7.3)	1(0.7)	2(1.3)	4.5(0.5–30.0)	12(0.5–36.0)
Fever	4(2.7)	3(2.0)	1(0.7)	1(0.7)	3.75(2.5–6.0)	6.75(2.0–12.0)
Chill	4(2.7)	4(2.7)	NA	NA	2.0(1.5–4.5)	1.25(0.5–2.0)
Rash	NA	NA	NA	NA		
Pruritus	NA	NA	NA	NA		
Arthralgia/myalgia	3(2.0)	3(2.0)	NA	NA	12.0(8.0–20.0)	24.0(12.0–36.0)
Fatigue	3(2.0)	3(2.0)	NA	NA	6.0(4.0–12.0)	18.0(12.0–24.0)
Anorexia	3(2.0)	3(2.0)	NA	NA	20.0(6.0–30.0)	20.0(12.0–36.0)
Nausea/vomiting	1(0.7)	1(0.7)	NA	NA	1.5(NA)	12.0(NA)
Allergic reaction	NA	NA	NA	NA		
Hypertension	1(0.7)	1(0.7)	NA	1(0.7)	0.5(NA)	1.5(NA)
Pneumonitis	NA	NA	NA	NA		
Hepatitis	NA	NA	NA	NA		
Colitis	NA	NA	NA	NA		

Abbreviations: *NA* Not applicable

### Adjuvant CIK cell immunotherapy improves the prognosis of patients

**Fig. 3:**
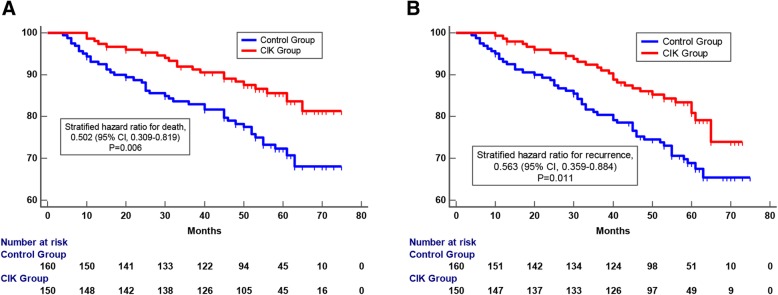
Survival analysis of postoperative breast cancer patients who received adjuvant CIK cell treatment (CIK treatment group, *n* = 150) compared to those who did not have CIK cell treatment (control group, *n* = 160). **a** Overall survival (OS) curves and (**b**) Recurrence-free survival (RFS) curves. Significantly improved prognosis was observed in the CIK treatment group compared to the control group. The Kaplan-Meier method was used to compare the survival rates, which were analyzed with the log-rank test

**Fig. 4:**
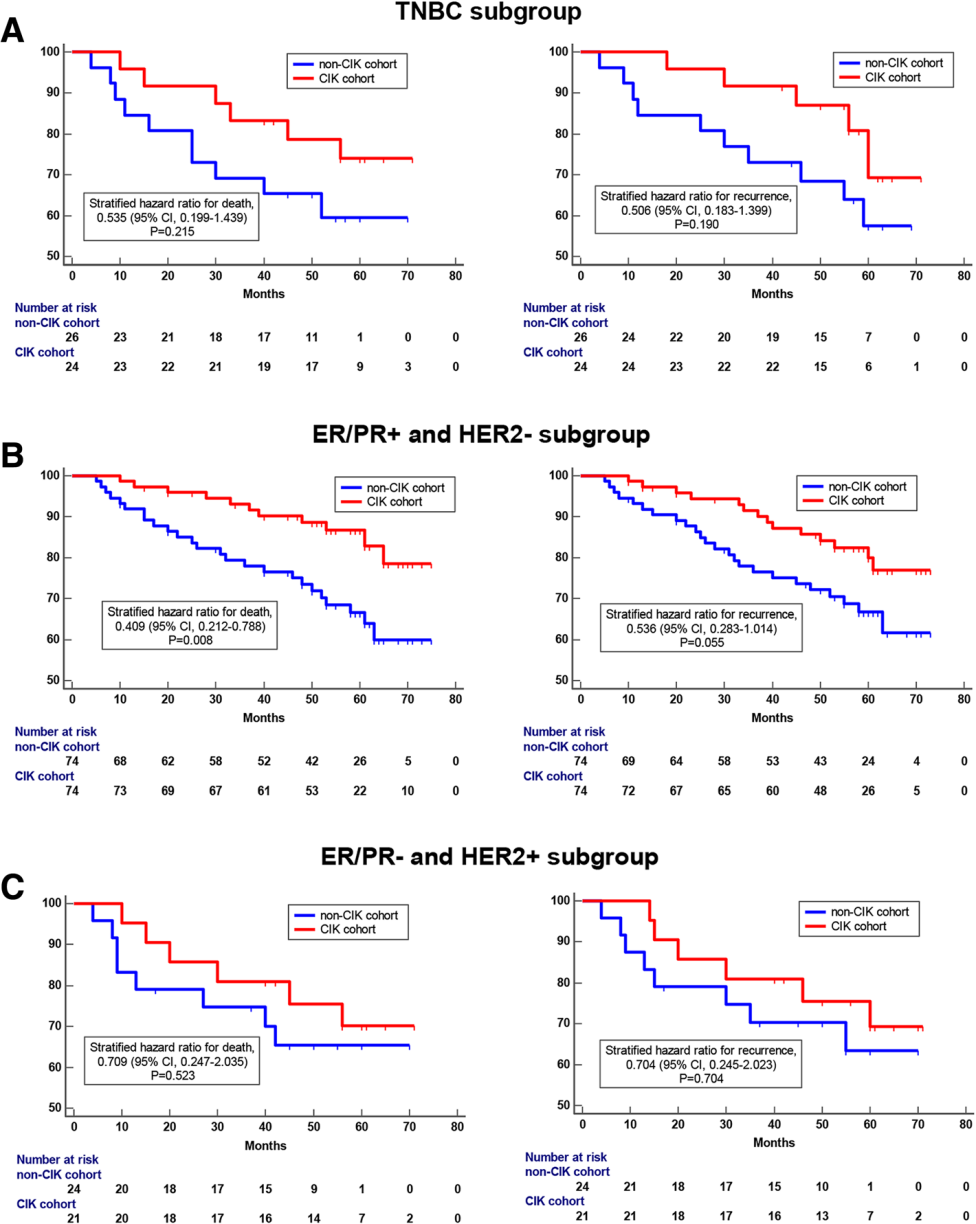
Kaplan-Meier curves of postoperative breast cancer patients in key subgroups. **a** OS and RFS curves of patients who received CIK treatment compared to those who did not in the TNBC subgroup (**b**) OS and RFS curves of patients who received adjuvant CIK cell treatment compared to those who did not in the ER/PR+ and HER2- breast cancer subgroup. **c** OS and RFS curves of patients who received adjuvant CIK cell treatment compared to those who did not in the ER/PR- and HER2+ subgroup

Survival analysis showed that patients had significantly better OS rates and RFS rates in the CIK treatment group than that in the control group (Fig. 3a and b). The 5-year OS rates and 5-year RFS rates for patients in the CIK treatment group were 85.7 and 80.8%, respectively, compared with 72.3 and 68.6% for patients in the control group, respectively. It was obvious that adjuvant CIK cell immunotherapy could improve the prognosis of postoperative breast cancer patients. Further, survival analysis was performed for some key subgroups of breast cancer. In the triple-negative breast cancer (TNBC) subgroup, patients were also found to benefit from the adjuvant CIK cell immunotherapy, however, due to limitations of sample size (total number of patients was 50, including 24 in CIK treatment group and 26 in control group), this benefit was not statistically significant (Fig. 4a). In the ER/PR+ and HER2- subgroup, CIK adjuvant treatment significantly prolonged the overall survival of patients (Fig. 4b). In the ER/PR- and HER2+ subgroup, CIK therapy also had a potential value in improving prognosis, however, due to the limited number of patients, it was not statistically significant for prolonging OS or RFS (Fig. 4c). In addition, all the breast cancer patients performed routine blood tests before and after 1–4 cycle of CIK infusion. We found that there were no obvious changes in the numbers of peripheral blood lymphocytes of the patients before and after each cycle (1, 2, 3, and 4) of CIK infusion (Additional file 2: Figure S2).

### Patterns and quantification of PD-L1 expression in breast cancer tissue

**Fig. 5:**
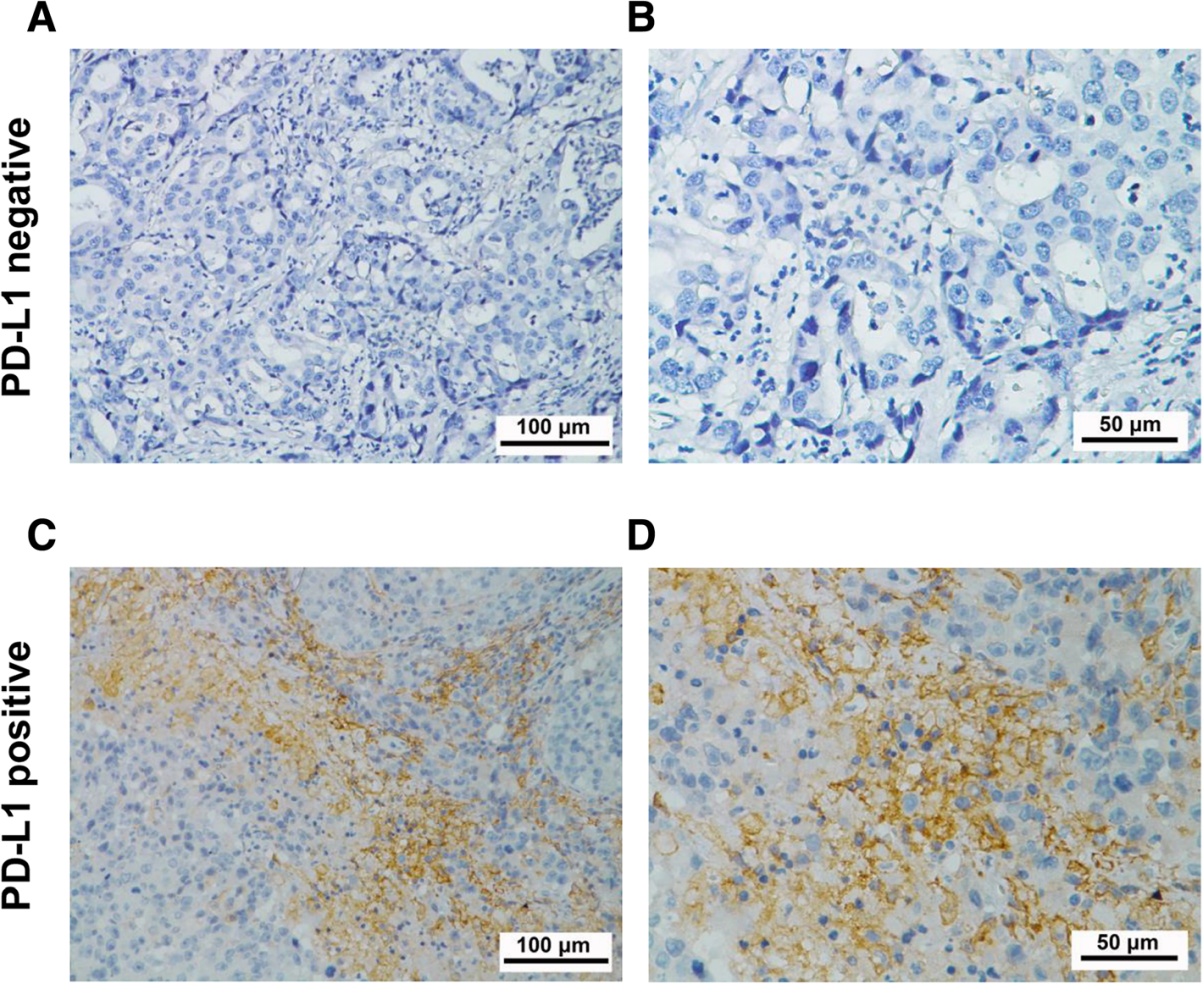
Immunohistochemical analysis of PD-L1 expression in surgical breast cancer specimens. Positive cases are determined based on the percentage of tumor cells with membranous PD-L1 staining. **a**, **b** PD-L1-negative expression and (**c**, **d**) PD-L1-positive expression. PD-L1 staining is shown by the brown chromogen. (**a** and **c**, 200×magnification; **b** and **d**, 400×magnification)

Immunohistochemical staining showed that PD-L1 was predominantly expressed on the cell membrane of breast tumor cells (Fig. 5c and d). In this study, we defined membranous PD-L1 staining in over 5% of tumor cells as positive according to the criteria previously described in similar study [28]. The number of PD-L1-positive cases was 86 (27.7%) among all breast cancer tissue samples: the control group contained 42 positive cases (26.3%) and the CIK treatment group contained 44 positive cases (29.3%) (Table 1).

### Associations between PD-L1 expression and survival benefits from CIK cell therapy

To explore the potential factors that affect the clinical efficacy of CIK treatment, we conducted univariate and multivariate Cox proportional hazards regression analyses in patients who received adjuvant CIK treatment. We included several clinicopathological parameters into the Cox regression analysis, such as age, TNM staging, positive lymph node ratio, pathological grade and PD-L1 expression. The univariate analysis results showed that tumor size, TNM stage, Herb2 expression and PD-L1 expression contributed to the outcomes of adjuvant CIK therapy. In the multivariate analysis, TNM stage and PD-L1 expression were independent prognostic factors for patients who received CIK therapy (Tables 3 and 4).

**Table 3:** Univariate and multivariate analysis of overall survival (OS) for breast cancer patients who received adjuvant CIK cell immunotherapy

Variables	Univariate analysis			Multivariate analysis		
	HR	95% CI	*p* value	HR	95% CI	*p* value
Age (< 50 vs. ≥ 50)	1.031	0.670–1.231	0.747			
Tumor size (< 20 vs. ≥ 20) (mm)	2.918	1.554–4.609	< 0.001*	1.895	0.881–2.805	0.493
TNM stage (I-II vs. III)	1.732	1.275–2.618	0.013*	1.787	1.271–2.991	0.015*
Histological differentiation (I-II vs. III)	1.078	0.487–1.499	0.718			
Positive lymph node ratio (< 0.21 vs. ≥ 0.21)	2.004	0.812–2.260	0.083			
ER (pos vs. neg)	0.714	0.513–1.919	0.057			
PR (pos vs. neg)	0.721	0.486–1.542	0.501			
Herb2 (pos vs. neg)	1.405	1.207–2.711	0.029*	1.193	0.814–2.569	0.807
PD-L1 expression (pos vs. neg)	0.610	0.352–0.903	< 0.001*	0.569	0.318–0.830	< 0.001*
TNBC (yes vs. no)	1.902	0.890–2.925	0.604			

*HR* Hazard ratio, *CI* Confidence interval, *Statistically significant, *p* <  0.05

**Table 4:** Univariate and multivariate analysis of recurrence-free survival (RFS) for breast cancer patients who received adjuvant CIK cell immunotherapy

Variables	Univariate analysis			Multivariate analysis		
	HR	95% CI	*p* value	HR	95% CI	*p* value
Age (< 50 vs. ≥ 50)	1.084	0.723–1.359	0.689			
Tumor size (< 20 vs. ≥ 20) (mm)	2.970	1.668–4.515	< 0.001*	1.903	0.311–3.783	0.546
TNM stage (I-II vs. III)	1.803	1.389–3.019	0.020*	1.695	1.119–2.513	0.028*
Histological differentiation (I-II vs. III)	1.015	0.801–1.763	0.812			
Positive lymph node ratio (< 0.21 vs. ≥ 0.21)	1.997	0.873–3.072	0.065			
ER (pos vs. neg)	0.582	0.312–1.878	0.475			
PR (pos vs. neg)	0.718	0.453–2.145	0.327			
Herb2 (pos vs. neg)	1.510	1.108–2.918	0.035*	1.792	0.589–2.807	0.699
PD-L1 expression (pos vs. neg)	0.596	0.214–0.963	< 0.001*	0.604	0.437–0.895	< 0.001*
TNBC (yes vs. no)	1.958	0.698–3.217	0.760			

*HR* Hazard ratio, *CI* confidence interval; *Statistically significant, *p* < 0.05

**Fig. 6:**
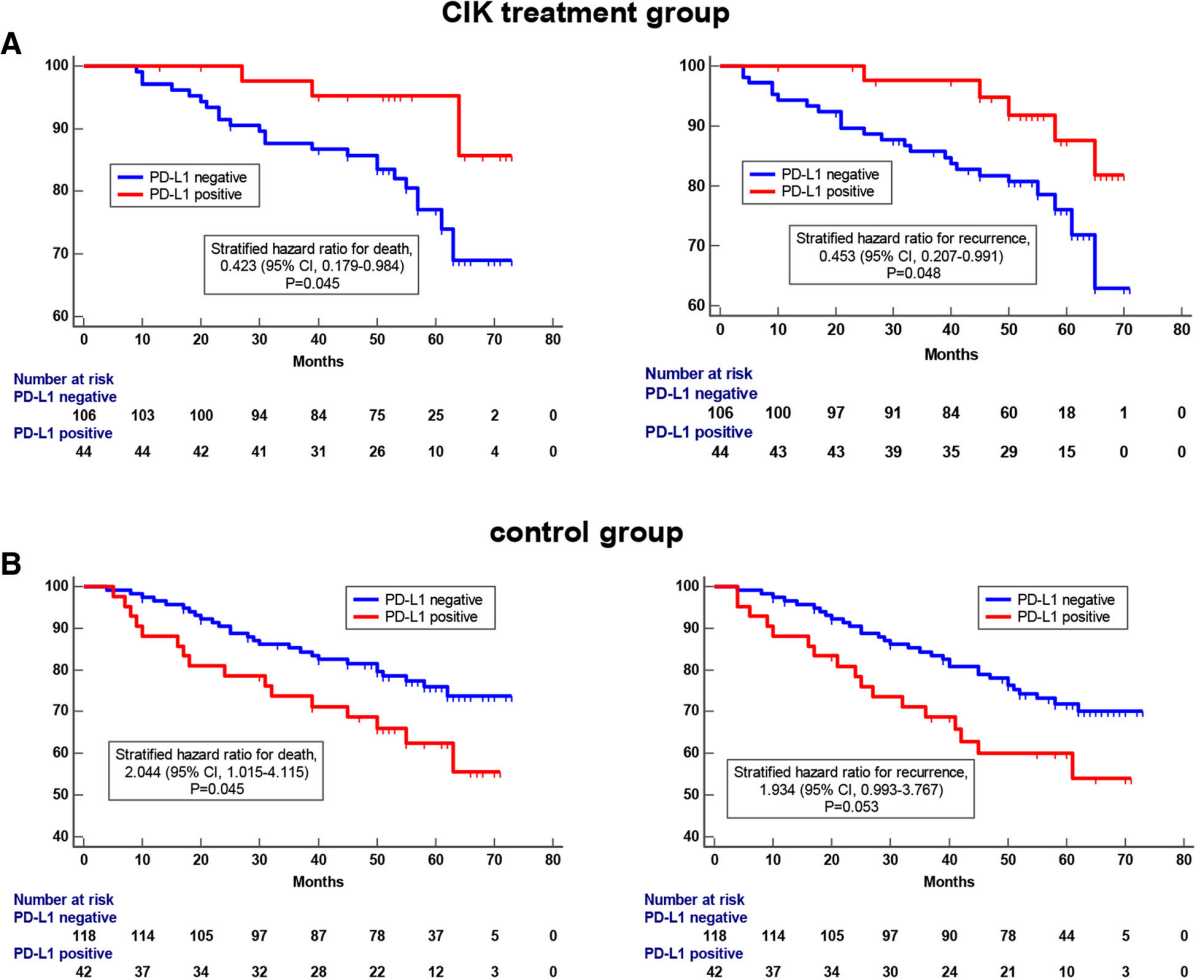
Kaplan-Meier curves of breast cancer patients based on postoperative treatment. **a** OS and RFS curves of patients in CIK treatment group. Significantly improved prognosis was observed in patients with PD-L1-positive expression. **b** OS and RFS curves of patients in the control group. Patients with PD-L1-negative expression had better prognosis than patients with PD-L1-positive expression

We next divided the patients in the CIK treatment group into two cohorts based on PD-L1 expression (PD-L1 positive vs. PD-L1 negative) and compared their survivals. Patients with PD-L1-positive expression tended to benefit from CIK treatment. In the PD-L1-positive cohort, the 5-year OS rate of patients was 95.2%, and the 5-year RFS rate was 87.6%. In the PD-L1-negative cohort, the 5-year OS rate and the 5-year RFS rate were 77.1 and 76.4%, respectively (Fig. 6a). We also stratified the patients in the control group based on PD-L1 expression to compare survival. However, patients with positive PD-L1 expression exhibited worse 5-years OS compared to those with negative PD-L1 expression in this group (Fig. 6b), which was consistent with previous studies [28]. Notably, in both of the control group and the CIK treatment group, the clinicopathological parameters between the internal two cohorts (PD-L1 positive vs. PD-L1 negative) were well matched, and there were no statistically significant differences in variables (Additional file 3: Table S1).

### PD-L1 expression is predictive of clinical benefit from adjuvant CIK cell-based treatment among patients with breast cancer

**Fig. 7:**
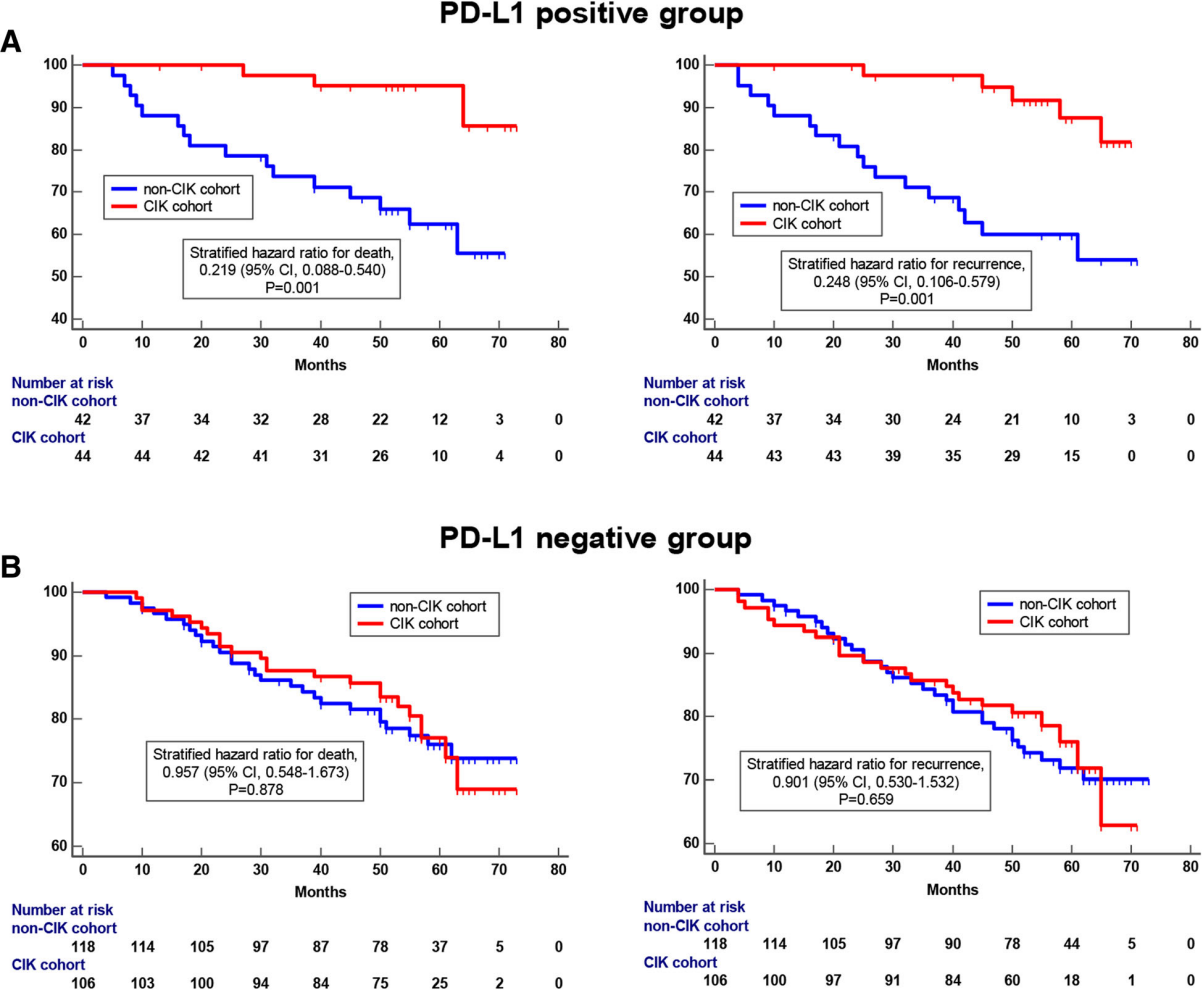
Kaplan-Meier curves of OS and RFS for breast cancer patients based on the expression of PD-L1 on tumor cells. **a** Survival differences between patients who received CIK treatment and patients who did not have CIK treatment in the PD-L1-positive cohort; (**b**) OS and RFS curves of patients who received CIK treatment and patients who did not have CIK treatment in the PD-L1-negative cohort

Based on the above findings, we supposed that tumor PD-L1 expression could be used as a biomarker for adjuvant CIK therapy in postoperative breast cancer patients. To address this possibility, we divided all the patients who were enrolled in this study (including the control group and the CIK treatment group) into two cohorts based on PD-L1 expression (PD-L1 positive vs. PD-L1 negative). In each cohort, we compared the difference in prognosis between patients treated with and without adjuvant CIK therapy. In the PD-L1-positive cohort, patients who received CIK treatment had better OS rates and RFS rates than patients who did not receive CIK treatment (Fig. 7a). Interestingly, in the PD-L1-negative cohort, there was no significant difference in prognosis regardless of whether patients received CIK treatment (Fig. 7b). These data indicated that breast cancer patients with PD-L1 tumor expression were more likely to benefit from adjuvant CIK cell immunotherapy.

## Discussion

Consistent with previous studies on other cancer types that have demonstrated that CIK cell infusion reduces tumor recurrence and prolongs patient survival [16, 17, 20, 21], our study validated the clinical benefits of adjuvant CIK immunotherapy for postoperative breast cancer patients, including TNBC patient. Importantly, we focused our research on the relationship between characteristics of the immune microenvironment and clinical benefit of breast cancer patients from adjuvant CIK immunotherapy. We explored whether PD-L1 expression could also serve as a predictor of adjuvant CIK therapy among breast cancer patients after complex treatment. In this study, we found that PD-L1 is mainly expressed in the cell membrane of breast cancer cells. Based on the measures used in previous literatures and the actual PD-L1 staining patterns, we made 5% tumor cell membrane expression as the threshold for PD-L1-positive expression. The Cox proportional regression analyses showed that PD-L1 expression was an independent prognostic factor for postoperative CIK treatment. In addition, when 5% was used as a stratification standard to distinguish all the patients, people who received CIK cell infusion had prolonged OS and RFS in the PD-L1 ≥ 5% expression cohort. Therefore, we think that over 5% PD-L1 tumor expression can be used as a predictor of CIK-assisted immunotherapy for postoperative breast cancer patients after comprehensive treatment.

The tumor development and progression are closely correlated with the interaction between the tumor microenvironment and tumor cells. PD-L1 is an important immunosuppressive molecule that can bind to its ligand PD-1 on tumor antigen-specific T cells. The engagement of PD-1/PD-L1 can mediate the disability of major histocompatibility complex (MHC)-restricted T cells, thereby inhibiting effective anti-tumor immune function [24, 25]. For this reason, PD-L1 is well known as a poor prognostic indicator for multiple tumors. Qin et al. indicated that breast cancer patients with higher PD-L1 expression had an approximately 2-fold higher risk of tumor recurrence, metastasis and cancer-related death [28]. In our study, this immune resistance also explains why patients with higher PD-L1 expression in the control group had a worse prognosis.

In fact, overexpression of PD-L1 on tumor cells is the product of adaptive immune resistance, which reflects the ongoing anti-tumor immunity in vivo. Immune resistance occurs when cancer cells change their phenotype in response to a cytotoxic or pro-inflammatory immune response, thereby evading the immune attack [30, 36]. Specifically, when T cells recognize tumor cells and release immune-activating cytokines, cancers can upregulate PD-L1 expression to limit the anti-tumor actions and protect themselves from T cells [31]. It has reported that PD-L1 upregulation is mainly induced by activated CD8^+^ cytotoxic T cells that are already present in the milieu rather than by constitutive expression in the HCC tumor cells [37]. Laurence et al. also revealed that PD-1/PD-L1 expression were associated with higher tumor-infiltrating lymphocytes densities in breast tumors [38]. These facts show us that patients with high PD-L1 expression are more likely to recruit immune cells to the cancer nests, which have a better anti-tumor immune status, so that the infused CIK cells were more likely to migrate to the tumor sites. Unfortunately, however, due to the lack of tumor tissue samples from patients after completion of CIK reinfusion, we cannot intuitively observe the increased immune cell infiltration within the tumors.

Adaptive immune resistance provides a strong theoretical basis for the clinical efficacy of PD-1- or PD-L1-blocking antibodies [39], which are able to reactivate an anti-tumor immune response from MHC-restricted T cells through the inhibition of immunological checkpoints [40]. A phase 3 clinical trial, IMpassion130, also revealed the benefit of combining anti-PD-L1 or anti-PD-1 antibody with standard chemotherapy for the first line treatment of metastatic TNBC, in which, the clinical benefit was particularly notable in the PD-L1 positive cohort [41]. However, we should not only focus on the straightforward disruption of the PD-1/PD-L1 suppression axis, it is considerable that strengthening the MHC non-restrictive immunity to supplement and strengthen anti-tumor immunity. CIK cell immunotherapy is well-suited to achieve the abovementioned effects and provides additional anti-tumor ability to patients who have developed adaptive immune resistance. CIK cell-based immunotherapy disrupts the MHC-mediated restriction and kills tumor cells in three ways: *a.* direct-killing: CIK cells can recognize tumor cells through different mechanisms and release toxic particles (such as granzyme and perforin), resulting in tumor cell lysis; *b.* a large release of inflammatory cytokines (such as IFN-γ, TNF-α and IL-2): these cytokines have a direct inhibitory effect on tumor cells and kill tumor cells by regulating immune system reactivity in vivo; and *c*. CIK cells induce tumor cell apoptosis: CIK cells can express Fas-L during culture and induce tumor cell apoptosis by binding to its ligand Fas which is expressed on the tumor cell membrane [12–14, 42]. In this study, the observation that patients with high PD-L1 expression were easier to benefit from postoperative CIK immunotherapy confirms that CIK cell infusion can ameliorate the immune anergy and provide additional immune function. Thus, the expression level of PD-L1 in the tumor is not only a screening indicator for PD-1/PD-L1 antibody therapy but may also be relevant for the development of CIK immunotherapy. In addition, whether combination therapy of PD-1/PD-L1 monoclonal antibody and CIK treatment can strengthen anti-tumor immunity and synergistically improve the prognosis of cancer patients requires confirmation by further preclinical and clinical research.

## Conclusions

We confirmed that CIK immunotherapy could improve the prognosis of breast cancer patients and for the first time revealed that PD-L1 expression in the tumor is as an indicator of adjuvant CIK therapy for postoperative breast cancer. Importantly, our findings on the relationship between PD-L1 expression and CIK therapy would provide new insights into the theory of tumor immunotherapy. Additional multicenter and large-sample validation studies are required to verify our results.

## Additional files

Additional file 1: **Figure S1.** CIK cell treatment protocol. (PDF 306 kb)

Additional file 2: **Figure S2.** The numbers of peripheral blood lymphocytes of the patients before and after each cycle (1, 2, 3, and 4) of CIK infusion. NS, not significant. (PDF 78 kb)

Additional file 3: **Table S1.** Demographics and clinical characteristics of patients with high/low PD-L1 expression. (PDF 157 kb)

## Data Availability

All data analyzed are included in this article and additional information is available upon request.

## Abbreviations

CIK cell: Cytokine induced killer cell
HCC: Hepatocellular carcinoma
OS: Overall survival
PBMC: Peripheral blood mononuclear cells
RFS: Recurrence-free survival
TNBC: Triple-negative breast cancer
TNM staging: Tumor–Node–Metastasis staging

## References

[1] Parkin DM, Bray F, Ferlay J, Pisani P (2005). Global cancer statistics, 2002. CA Cancer J Clin.

[2] Anderson BO, Yip CH, Ramsey SD, Bengoa R, Braun S, Fitch M (2006). Breast cancer in limited-resource countries: health care systems and public policy. Breast J.

[3] Ferlay J, Shin HR, Bray F, Forman D, Mathers C, Parkin DM (2010). Estimates of worldwide burden of cancer in 2008: GLOBOCAN 2008. Int J Cancer.

[4] Lukong KE, Ogunbolude Y, Kamdem JP (2017). Breast cancer in Africa: prevalence, treatment options, herbal medicines, and socioeconomic determinants. Breast Cancer Res Treat.

[5] Berry DA, Cronin KA, Plevritis SK, Fryback DG, Clarke L, Zelen M (2005). Effect of screening and adjuvant therapy on mortality from breast cancer. N Engl J Med.

[6] Davies C, Pan H, Godwin J, Gray R, Arriagada R, Raina V (2013). Long-term effects of continuing adjuvant tamoxifen to 10 years versus stopping at 5 years after diagnosis of oestrogen receptor-positive breast cancer: ATLAS, a randomised trial. Lancet..

[7] Haynes B, Sarma A, Nangia-Makker P, Shekhar MP (2017). Breast cancer complexity: implications of intratumoral heterogeneity in clinical management. Cancer Metastasis Rev.

[8] Linos E, Spanos D, Rosner BA, Linos K, Hesketh T, Qu JD (2008). Effects of reproductive and demographic changes on breast cancer incidence in China: a modeling analysis. J Natl Cancer Inst.

[9] Giancola R, Olioso P, Di Riti M, Capone A, Contento A, Pompetti F (2008). Evaluation of an automated closed fluid management device for processing expanded cytokine-induced killer cells to use in immunotherapy programs for cancer. Transfusion..

[10] Schmidt-Wolf IG, Negrin RS, Kiem HP, Blume KG, Weissman IL (1991). Use of a SCID mouse/human lymphoma model to evaluate cytokine-induced killer cells with potent antitumor cell activity. J Exp Med.

[11] Zoll B, Lefterova P, Csipai M, Finke S, Trojaneck B, Ebert O (1998). Generation of cytokine-induced killer cells using exogenous interleukin-2, −7 or −12. Cancer Immunol Immunother.

[12] Linn YC, Hui KM (2003). Cytokine-induced killer cells: NK-like T cells with cytotolytic specificity against leukemia. Leuk Lymphoma.

[13] Nagaraj S, Ziske C, Schmidt-Wolf IG (2004). Human cytokine-induced killer cells have enhanced in vitro cytolytic activity via non-viral interleukin-2 gene transfer. Genet Vaccines Ther.

[14] Gao X, Mi Y, Guo N, Xu H, Xu L, Gou X, et al. Cytokine-induced killer cells as pharmacological tools for Cancer immunotherapy. Front Immunol. 2017;8(774) 10.3389/fimmu.2017.00774. 10.3389/fimmu.2017.00774 PMC5498561 28729866

[15] Chan JK, Hamilton CA, Cheung MK, Karimi M, Baker J, Gall JM (2006). Enhanced killing of primary ovarian cancer by retargeting autologous cytokine-induced killer cells with bispecific antibodies: a preclinical study. Clin Cancer Res.

[16] Jakel CE, Schmidt-Wolf IG (2014). An update on new adoptive immunotherapy strategies for solid tumors with cytokine-induced killer cells. Expert Opin Biol Ther.

[17] Linn YC, Lau LC, Hui KM (2002). Generation of cytokine-induced killer cells from leukaemic samples with in vitro cytotoxicity against autologous and allogeneic leukaemic blasts. Br J Haematol.

[18] Liu L, Zhang W, Qi X, Li H, Yu J, Wei S (2012). Randomized study of autologous cytokine-induced killer cell immunotherapy in metastatic renal carcinoma. Clin Cancer Res.

[19] Ma Y, Zhang Z, Tang L, Xu YC, Xie ZM, Gu XF (2012). Cytokine-induced killer cells in the treatment of patients with solid carcinomas: a systematic review and pooled analysis. Cytotherapy..

[20] Marten A, Renoth S, von Lilienfeld-Toal M, Buttgereit P, Schakowski F, Glasmacher A (2001). Enhanced lytic activity of cytokine-induced killer cells against multiple myeloma cells after co-culture with idiotype-pulsed dendritic cells. Haematologica..

[21] Sangiolo D, Mesiano G, Gammaitoni L, Leuci V, Todorovic M, Giraudo L (2014). Cytokine-induced killer cells eradicate bone and soft-tissue sarcomas. Cancer Res.

[22] Flieger D, Kufer P, Beier I, Sauerbruch T, Schmidt-Wolf IG (2000). A bispecific single-chain antibody directed against EpCAM/CD3 in combination with the cytokines interferon alpha and interleukin-2 efficiently retargets T and CD3+CD56+ natural-killer-like T lymphocytes to EpCAM-expressing tumor cells. Cancer Immunol Immunother.

[23] Gammaitoni L, Giraudo L, Macagno M, Leuci V, Mesiano G, Rotolo R (2017). Cytokine-induced killer cells kill chemo-surviving melanoma Cancer stem cells. Clin Cancer Res.

[24] Sharpe AH, Wherry EJ, Ahmed R, Freeman GJ (2007). The function of programmed cell death 1 and its ligands in regulating autoimmunity and infection. Nat Immunol.

[25] Fife BT, Pauken KE, Eagar TN, Obu T, Wu J, Tang Q (2009). Interactions between PD-1 and PD-L1 promote tolerance by blocking the TCR-induced stop signal. Nat Immunol.

[26] Deng C, Li Z, Guo S, Chen P, Chen X, Zhou Q (2017). Tumor PD-L1 expression is correlated with increased TILs and poor prognosis in penile squamous cell carcinoma. Oncoimmunology..

[27] Mittendorf EA, Philips AV, Meric-Bernstam F, Qiao N, Wu Y, Harrington S (2014). PD-L1 expression in triple-negative breast cancer. Cancer Immunol Res.

[28] Qin T, Zeng YD, Qin G, Xu F, Lu JB, Fang WF (2015). High PD-L1 expression was associated with poor prognosis in 870 Chinese patients with breast cancer. Oncotarget..

[29] Thompson RH, Kuntz SM, Leibovich BC, Dong H, Lohse CM, Webster WS (2006). Tumor B7-H1 is associated with poor prognosis in renal cell carcinoma patients with long-term follow-up. Cancer Res.

[30] Ribas A (2015). Adaptive immune resistance: how Cancer protects from immune attack. Cancer Discov.

[31] Tumeh PC, Harview CL, Yearley JH, Shintaku IP, Taylor EJ, Robert L (2014). PD-1 blockade induces responses by inhibiting adaptive immune resistance. Nature..

[32] Herbst RS, Soria JC, Kowanetz M, Fine GD, Hamid O, Gordon MS (2014). Predictive correlates of response to the anti-PD-L1 antibody MPDL3280A in cancer patients. Nature..

[33] Smit EF, van den Heuvel MM (2016). PD-L1 in non-small-cell lung cancer: the third target for immunotherapy. Lancet..

[34] Chen CL, Pan QZ, Zhao JJ, Wang Y, Li YQ, Wang QJ (2016). PD-L1 expression as a predictive biomarker for cytokine-induced killer cell immunotherapy in patients with hepatocellular carcinoma. Oncoimmunology..

[35] Weng DS, Zhou J, Zhou QM, Zhao M, Wang QJ, Huang LX (2008). Minimally invasive treatment combined with cytokine-induced killer cells therapy lower the short-term recurrence rates of hepatocellular carcinomas. J Immunother.

[36] Dondero A, Pastorino F, Della Chiesa M, Corrias MV, Morandi F, Pistoia V (2016). PD-L1 expression in metastatic neuroblastoma as an additional mechanism for limiting immune surveillance. Oncoimmunology..

[37] Xie QK, Zhao YJ, Pan T, Lyu N, Mu LW, Li SL (2016). Programmed death ligand 1 as an indicator of pre-existing adaptive immune responses in human hepatocellular carcinoma. Oncoimmunology..

[38] Buisseret L, Garaud S, de Wind A, Van den Eynden G, Boisson A, Solinas C (2017). Tumor-infiltrating lymphocyte composition, organization and PD-1/ PD-L1 expression are linked in breast cancer. Oncoimmunology..

[39] Powles T, Eder JP, Fine GD, Braiteh FS, Loriot Y, Cruz C (2014). MPDL3280A (anti-PD-L1) treatment leads to clinical activity in metastatic bladder cancer. Nature..

[40] Parsa AT, Waldron JS, Panner A, Crane CA, Parney IF, Barry JJ (2007). Loss of tumor suppressor PTEN function increases B7-H1 expression and immunoresistance in glioma. Nat Med.

[41] Schmid P, Adams S, Rugo HS, Schneeweiss A, Barrios CH, Iwata H (2018). Atezolizumab and nab-paclitaxel in advanced triple-negative breast Cancer. N Engl J Med.

[42] Alvarnas JC, Linn YC, Hope EG, Negrin RS (2001). Expansion of cytotoxic CD3+ CD56+ cells from peripheral blood progenitor cells of patients undergoing autologous hematopoietic cell transplantation. Biol Blood Marrow Transplant.

