# Supplementary figures and images for: PD-L1 expression is a predictive biomarker for CIK cell-based immunotherapy in postoperative patients with breast cancer
Source: J Immunother Cancer. 2019 Aug 27;7:228. doi: 10.1186/s40425-019-0696-8 (PMC6712838; doi:10.1186/s40425-019-0696-8)

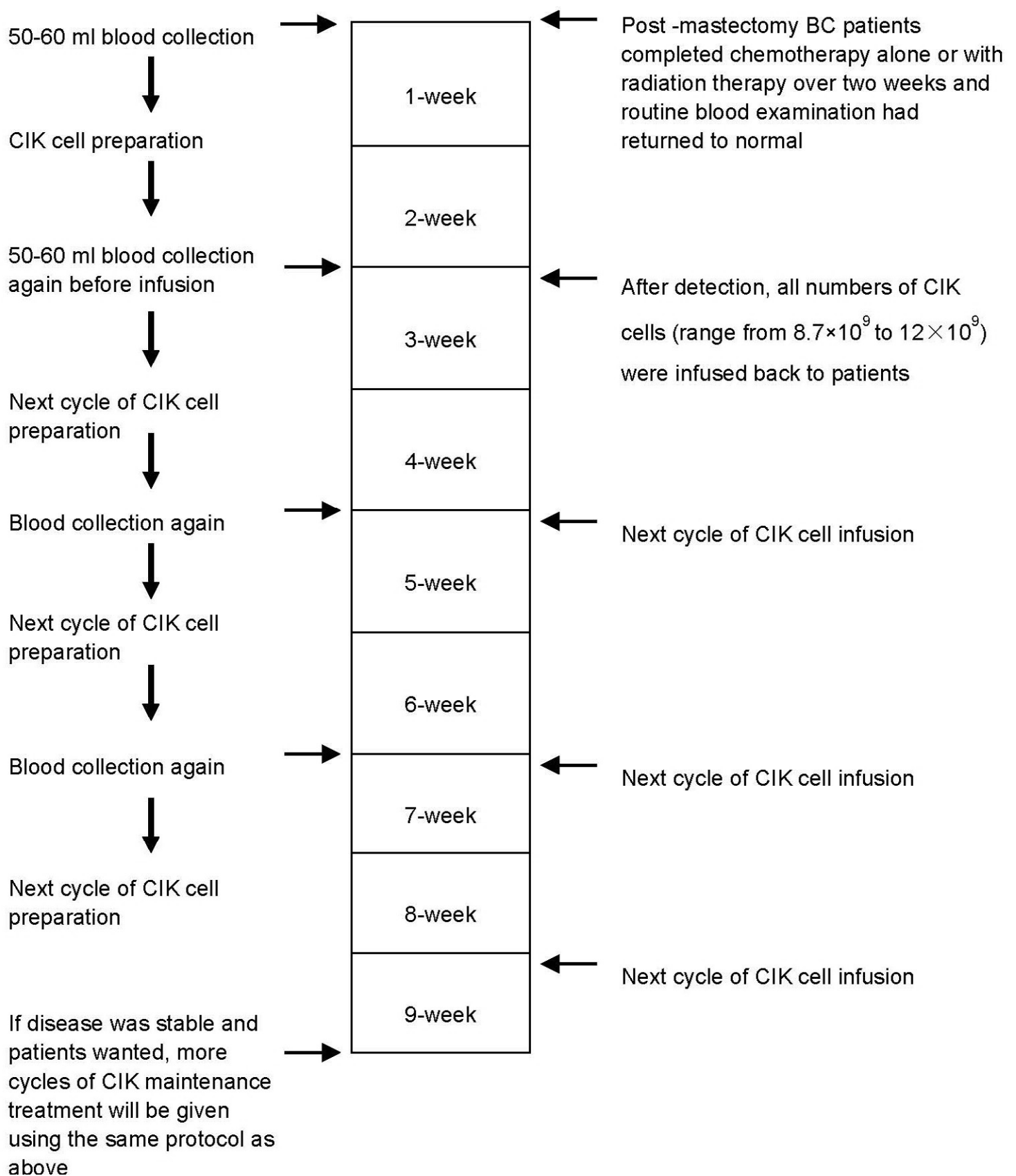

Supplement: Supplementary file 1 — Figure S1. CIK cell treatment protocol. (PDF 306 kb) [file 40425_2019_696_MOESM1_ESM.pdf]

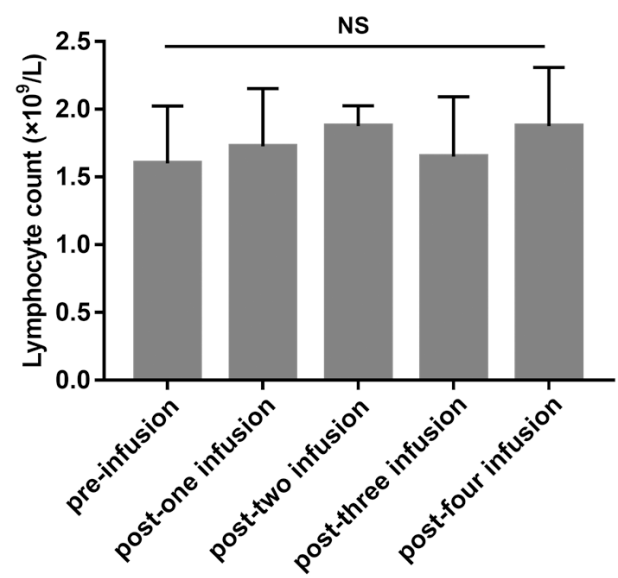

Supplement: Supplementary file 2 — Figure S2. The numbers of peripheral blood lymphocytes of the patients before and after each cycle (1, 2, 3, and 4) of CIK infusion. NS, not significant. (PDF 78 kb) [file 40425_2019_696_MOESM2_ESM.pdf]
