# Supplementary material for: PD-L1 expression is a predictive biomarker for CIK cell-based immunotherapy in postoperative patients with breast cancer
Source: J Immunother Cancer. 2019 Aug 27;7:228. doi: 10.1186/s40425-019-0696-8 (PMC6712838; doi:10.1186/s40425-019-0696-8)
Supplement: Supplementary file 3 — Table S1. Demographics and clinical characteristics of patients with high/low PD-L1 expression. (PDF 157 kb) [file 40425_2019_696_MOESM3_ESM.pdf]

Table S1. Demographics and clinical characteristics of patients with high/low PD-L1 expression.

Clinicopathologic variables	Control Group			CIK Treatment Group		
	High PD-L1 expression (n = 42)	Low PD-L1 expression (n = 118)	*p* value	High PD-L1 expression (n = 44)	Low PD-L1 expression (n = 106)	*p* value
**Age (y)**			0.580			0.158
**< 50**	14	45		19	33	
**≥ 50**	28	73		25	73	
**Tumor size (mm)**			0.288			0.882
**< 20**	17	59		23	54	
**≥ 20**	25	59		21	52	
**TNM stage**			0.880			0.934
**I**	5	14		5	10	
**II**	16	50		19	46	
**III**	21	54		20	50	
**Histological differentiation**			0.769			0.489
**I/II**	26	70		28	61	
**III**	16	48		16	45	
**Positive lymph node ratio**			0.957			0.984
**< 0.21**	16	43		15	35	
**0.21 ≤ x < 0.65**	21	59		22	53	
**≥ 0.65**	5	16		7	18	
**Receptor status**						
**ER**			0.881			0.637
**Positive**	13	38		14	38	
**Negative**	29	80		30	68	
**PR**			0.333			0.420
**Positive**	17	58		18	51	
**Negative**	25	60		26	55	
**Her2**			0.781			0.510
**Positive**	15	45		17	35	
**Negative**	27	73		27	71	
**Chemotherapy**			0.781			0.731
**Yes**	39	111		41	97	
**No**	3	7		3	9	
**Radiotherapy**			0.134			0.826
**Yes**	37	112		42	102	

**No**	5	6		2	4	
**Endocrinetherapy**			0.534			0.884
**Yes**	27	82		26	64	
**No**	15	36		18	42	
**TNBC**			0.932			0.639
**Yes**	7	19		8	16	
**No**	35	99		36	90	
